# Suppression of Connexin 43 Leads to Strial Vascular Hyper-Permeability, Decrease in Endocochlear Potential, and Mild Hearing Loss

**DOI:** 10.3389/fphys.2020.00974

**Published:** 2020-08-14

**Authors:** Jinhui Zhang, Xiaohan Wang, Zhiqiang Hou, Lingling Neng, Jing Cai, Yunpei Zhang, Xiaorui Shi

**Affiliations:** ^1^ Oregon Hearing Research Center, Oregon Health & Science University, Portland, OR, United States; ^2^ Boston Children’s Hospital, Harvard Medical School, Boston, MA, United States

**Keywords:** mouse, connexin 43, blood-labyrinth-barrier, perivascular macrophage, endocochlear potential, hearing loss

## Abstract

**Objective**: Connexin 43 (Cx43) is a protein constituent of gap junctions (GJs) in various barrier cells, especially astrocytes and microglia of the blood-brain-barrier (BBB), where it plays an important role in intercellular communication and regulation of the barrier. Despite the importance of Cx43 in other blood barriers, not much attention has been paid to expression and function of Cx43 in the blood-labyrinth-barrier (BLB) of the stria vascularis in the cochlea.

**Methods**: We used multiple research approaches, including immunocytochemical staining, patch-clamp dye loading technique, real-time quantitative reverse transcription (RT)-PCR, western blot, measurement of endocochlear potential (EP) with an electrode through the scala media, and auditory brainstem response to test hearing function.

**Results**: We found Cx43 expressed in vascular endothelial cells (ECs) and perivascular resident macrophages (PVMs) in the stria vascularis of adult C57BL/6 mouse cochleae. In particular, we found Cx43 expressed in foot processes of PVMs at points of contact with the endothelium. Consistent with Cx43 expression *in vivo*, we also found Cx43 expressed in EC-EC and EC-PVM interfaces in a co-cultured cell line model. Using a patch-clamp dye loading technique, we demonstrated that Alexa Fluor® 568 dye injected into PVMs diffuses to connected neighboring ECs. The functional coupling between the ECs and PVMs is blocked by 18α-Glycyrrhetinic acid (18α-GA), a GJ blocker. Suppression of Cx43 with small interfering RNA (siRNA) *in vivo* significantly elevated hearing threshold and caused the EP to drop and the blood barrier to become more permeable. In further study, using *in vitro* primary EC cell line models, we demonstrated that suppression of Cx43 disrupts intercellular tight junctions (TJs) in the EC monolayer and increases endothelial monolayer permeability.

**Conculsion**: Taken together, these findings underscore the importance of Cx43 expression in the normal ear for maintaining BLB integrity, normal EP, and hearing function.

## Introduction

Gap junction (GJ) expression in the inner ear is essential for audition (Mammano, 2019). GJ proteins such as connexin 26 (Cx26) and Cx30 are widely distributed in cochlear epithelial cells (Jagger and Forge, 2015; Verselis, 2019). They play critical roles in hearing, including roles in cochlear development and in sustaining auditory function in the mature cochlea (Qu et al., 2012; Verselis, 2019). Mutations in connexins in the cochlear epithelium, in particular mutation of Cx26, is known to be a common cause of congenital hearing loss (Avraham, 2001; Liu et al., 2001; Hoang Dinh et al., 2009; Lee and White, 2009; Martinez et al., 2009; Sun et al., 2009; Hong et al., 2010; Jagger and Forge, 2015; Wingard and Zhao, 2015).

Connexin 43 (Cx43), encoded by the GJ alpha-1 (GJA1) gene, has also been identified in cochlear tissues, including in the stria vascularis, spiral ligament, organ of Corti, and supporting cells of the vestibular sensory epithelia and spiral ganglia (Forge et al., 2003; Suzuki et al., 2003; Nagy et al., 2004; Procacci et al., 2008; Figueroa and Duling, 2009; Liu et al., 2014). Mutation in Cx43 has been reported to be linked to non-syndromic hearing loss (Liu et al., 2001; Martinez et al., 2009; Hong et al., 2010; Wingard and Zhao, 2015). Yet, despite the Cx43 mutation shown involved in congenital hearing loss, the physiological role of Cx43 plays in hearing is largely unknown. This is particularly true regarding its role in the blood-labyrinth barrier (BLB) of the stria vascularis.

The stria vascularis is an ion-transporting tissue in the inner ear that plays an important role in maintaining tissue homeostasis and the endocochlear potential (EP; Wangemann and Schacht, 1996). The stria vascularis lines the scala media and comprises basal cells, intermediate cells, and marginal cells, as well as of the capillary network which perfuses it. Functionally, the stria vascularis produces endolymph and maintains cochlear homeostasis and the EP. The EP is the critical driving force necessary for the depolarization of hair cells (HCs) and is thus central to hearing function (Tasaki and Spyropoulos, 1959; Wangemann, 2002, 2006; Chen and Zhao, 2014). The BLB, a highly specialized capillary network in the stria vascularis, controls exchanges between blood and the intrastitial space in the cochlea (Shi, 2011, 2016). The barrier shields the inner ear from blood-born toxic substances and selectively passes ions, fluids, and nutrients to the cochlea, playing an essential role in maintenance of the cochlear homeostasis needed for hearing function (Juhn and Rybak, 1981; Juhn, 1988; Shi, 2016). Anatomically, the BLB comprises endothelial cells (ECs) in the strial microvasculature, elaborated tight and adherens junctions, pericytes (PCs), basement membrane (BM), and perivascular resident macrophages (PVMs), which together form a complex “cochlear-vascular unit” (Shi, 2010; Lapenna et al., 2018). Physical interactions between the ECs, PCs, and PVMs, as well as signaling between the cells, are critical for controlling vascular permeability and providing a functionally stable environment (Shi, 2011, 2016).

In the blood-brain-barrier (BBB) of the central nervous system (CNS), Cx43 is predominately expressed in vascular ECs and astrocytes and is especially found in foot process contacts with capillaries (Danesh-Meyer and Green, 2008; Chew et al., 2010; Boulay et al., 2015). The role that Cx43 plays in intercellular communication and regulation of barrier function (other than in the BLB) is widely reported (Naus et al., 1991; Winkler et al., 2011; Boulay et al., 2015). However, the role of Cx43 in the strial BLB has not yet been investigated. In the present study, we report, for the first time, Cx43 immunoreactivity in the strial BLB of adult mouse (C57BL/6) cochleae. We found that downregulation of Cx43 leads to hearing loss. Supression of CX43 also strongly affects BLB integrity, causing strial hyper-permeability and decrease in EP. Further study in primary EC cell line models revealed that the Cx43 has an effect on TJ protein expression in the endothelial barrier. Taken together, the results underscore the importance of Cx43 expression in the strial BLB for maintenance of BLB integrity, normal EP, and hearing function.

## Materials and Methods

### Animals

All mice used in this work were purchased from The Jackson Laboratory (Bar Harbor, ME, USA). C57BL/6J mice (stock number: 000664, 6–8 weeks old) were used in all *in vivo* experiments, excepting the B6.129P2(Cg)-Cx3cr1^tm1Litt^/J transgenic mice (stock number: 005582, 6–8 weeks old) used in the *in vivo* immunofluorescence experiments for the visualization of Cx43 expression in PVMs. All procedures in this study were reviewed and approved by the Institutional Animal Care and Use Committee (IACUC) at Oregon Health & Science University (IP 00000968).

### Immunohistochemistry

The procedure for immunohistochemistry was previously reported (Zhang et al., 2012). Briefly, mice were deeply anesthetized by intraperitoneal injection of a cocktail containing ketamine hydrochloride (100 mg/kg, Henry Schein, Melville, NY) and xylazine hydrochloride (40 mg/kg, Par Pharmaceutical Companies, Inc. Spring Valley, NY). After sedation, mice were perfused intravascularly through the left ventricle with phosphate-buffered saline (PBS, pH 7.4) to flush out circulating blood, followed by a 4% paraformaldehyde fixative. The mice were decapitated and the cochleae harvested and fixed in 4% paraformaldehyde overnight. Whole-mounts of stria vascularis were carefully isolated, washed in PBS, permeabilized in 0.5% Triton X-100 (T8787, Sigma-Aldrich, St. Louis, MO) in PBS for 0.5 h, and immuno-blocked with a solution of 1% fish gelatin (G7765, Sigma-Aldrich, St. Louis, MO) in PBS for an additional hour. The specimens were incubated overnight at 4°C with the primary antibody (Table 1), a rabbit polyclonal, to detect Cx43 (ab11370, Abcam, Cambridge, MA) in 1% bovine serum albumin (BSA) diluted in PBS. After three washes in PBS, samples were incubated with the secondary antibody, Alexa Fluor 568-conjugated goat anti-rabbit IgG (A-11011, Thermo Fisher Scientific, Waltham, MA), for 1 h at room temperature. Capillaries were labeled with the lectin Griffonia simplicifolia IB4 (GS-IB4) conjugated to Alexa Fluor 647 (I32450, Thermo Fisher Scientific, Waltham, MA). The tissues were washed three times and mounted in mounting medium (H-1500, Vector Laboratories, Burlingame, CA).

**Table 1:** Antibodies used in the study.

Antibodies	Vectors	Cat#	Dilution source (dilution with 1% BSA-PBS)		Application
ZO-1	Invitrogen	61-7300	1:25	Rabbit polyclonal for ZO-1	Labeling TJ associated protein
VE-cadherin	Abcam	Abcam	1:50	Rabbit polyclonal for VE-cadherin	Labeling TJ associated protein
Occludin	Abcam	Ab31721	1:50	Rabbit polyclonal for occludin	Labeling TJ associated protein
Connexin 43	Abcam	Ab11370	1:100	Rabbit polyclonal for connexin 43/GJA1	Labeling gap junction protein (in tissue)
Connexin 43	Abcam	Ab78055	1:250	Mouse monoclonal for connexin 43/GJA1	Labeling gap junction protein (in cell line)
von Willebrand Factor	Abcam	Ab11713	1:100	Sheep polyclonal for von Willebrand factor	Labeling endothelial cells

### EC Culture and EC-PVM Co-culture Staining for Cx43

Protocols for generating the cell lines used in these experiments were previously reported (Neng et al., 2013). Briefly, cochleae from 10- to 15-day-old C57BL/6J mice were harvested under sterile conditions. The stria vascularis was gently pulled away from the spiral ligament and placed in ice-cold perilymph solution containing 125 mmol/L NaCl, 3.5 mmol/L KCl, 1.3 mmol/L CaCl_2_, 1.5 mmol/L MgCl_2_, 0.51 mmol/L NaH_2_PO_4_, 10 mmol/L HEPES, and 5 mmol/L glucose at pH7.4 with osmolarity adjusted to 310 mmol/kg. The isolated stria vascularis was washed with gentle shaking in cool perilymph solution for 10 min and transferred to a clean 35-mm collagen I-coated dish with 2 ml of Endothelial Cell Medium (1001, ScienCell™ Research Laboratories, Carlsbad, CA) or Macrophage Medium (1921, ScienCell™ Research Laboratories, Carlsbad, CA). The stria vascularis was cut into small pieces with sterile ophthalmic tweezers under a dissection microscope. Fragmented pieces of the stria vascularis were seeded around the dish to roughly uniform density. The procedure for situating the fragments requires approximately 2 h. The medium is changed every 3 days. The cells are incubated at 37°C in 5% CO_2_ for 7–14 days until the cell clones melt. Cell clusters of each phenotype are visible by phase contrast microscopy at 6–7 days, at which time the cells are passaged into a 60-mm collagen I-coated dish. The cultured cells were purified by fluorescence-activated cell sorting. Purified primary PVMs were fluorescence encoded with green fluorescent protein (GFP), as previously described (Neng et al., 2015). Purified primary ECs and fluorescence tagged PVMs were cultured or co-cultured in separate dishes or in mixtures of the two cell types for 3 days. The cells were then fixed in 4% paraformaldehyde in PBS for 15 min at room temperature, permeabilized in 0.5% Triton X-100 in PBS for 5 min, blocked with 1% fish gelatin for 1 h, and incubated overnight at 4°C with the primary antibodies. The primary antibodies for the EC culture included anti-von Willebrand factor (vWF; ab11713, Abcam, Cambridge, MA) for ECs and anti-Cx43 for Cx43 diluted in 1% BSA in PBS. The primary antibody for the EC-PVM co-culture was only anti-Cx43 diluted in 1% BSA in PBS. After three washes in PBS, the samples were incubated with secondary antibodies, either Alexa Fluor 488-conjugated donkey anti-sheep IgG (A-11015, Thermo Fisher Scientific, Waltham, MA) or Alexa Fluor 568-conjugated goat anti-rabbit IgG for 1 h at room temperature. Images were acquired under an FV1000 Olympus laser-scanning confocal microscope (Olympus FV1000, Japan), saved as OIB files, and further processed using Adobe Photoshop CS2 (Adobe Systems, San Jose, CA).

### Dye Injection With a Patch-Clamp Pipette

The EC and PVM co-culture was bathed in artificial cerebrospinal fluid (ACSF) at room temperature on day 3. Pipettes for dye delivery were prepared using a micropipette puller (Sutter, P-97). When back-filled with the internal solution, the typical resistance of the pipette is about 15 mΩ in ACSF. The ACSF used to bath the cell line consists of: 137 mM sodium chloride, 0.5 mM sodium bicarbonate, 1 mM sodium phosphate, 3 mM potassium chloride, 2 mM calcium chloride, 1 mM magnesium sulfate, 20 mM HEPES, and 16 mM glucose, with the pH adjusted to 7.4 with sodium hydroxide. The internal solution contains: 125 mM potassium gluconate, 5 mM sodium chloride, 4 mM EGTA, 10 mM HEPES, 4 mM ATP-Mg, 0.5 mM GTP-Na, 10 mM phosphocreatine, and 1 mM magnesium chloride, with the pH adjusted to 7.2 with potassium hydroxide. Alexa 568 Hydrazide 35 μg/ml (A10437, Thermo Fisher Scientific, Waltham, MA) was added to the internal solution and dialyzed for 1 min. Alexa 488 Hydrazide (A10436, Thermo Fisher Scientific, Waltham, MA) was then delivered to the cells (PVMs) in whole-cell mode. Since the Alexa dye is negatively charged, a negative potential (−10 mV) was applied to expedite diffusion of the dye. To block the GJ, 18α-Glycyrrhetinic acid (18α-GA,10 μM, G8503, Sigma-Aldrich, St. Louis, MO) was added to the culture medium for 30 min (Zhu et al., 2016; Jiang et al., 2017).

### *In vivo* siRNA Transfection and Real-Time Quantitative RT-PCR

For the small interfering RNA (siRNA) transfection, mice were anesthetized, a 5 μl solution of Gja1 Silencer® Select Pre-designed siRNA (40 μM, 4390771, Assay ID: s66667, Thermo Fisher Scientific, Waltham, MA) was injected through the posterior-inferior quadrant (Qi et al., 2014) with a Hamilton’s microliter syringe (30 G needle), and the middle ear was filled completely with the solution for 3 days. Scrambled siRNA (4390846, Thermo Fisher Scientific, Waltham, MA) of the same concentration was given to the control group. This method was previously used and reported (Zhang et al., 2012). Three days following the injection, mice were sacrificed, and the cochleae were dissected. Total RNA was extracted from the stria vascularis.

The procedure for quantitative RT-PCR was previously described and reported (Yang et al., 2011). Briefly, a total RNA (300–500 ng) was extracted from control scrambled siRNA treated (*n* = 6) and Gja1 siRNA treated animals (*n* = 6) using an RNeasy MinElute Cleanup Kit (74204, Qiagen, Valencia, CA), as per the manufacturer’s recommendations. The sample for total RNA was reverse-transcribed with a RETROscript™ Reverse Transcription Kit (AM1710, Thermo Fisher Scientific, Waltham, MA). Complementary DNA (cDNA) synthesized from total RNA was diluted 10-fold with DNase-free water, with each cDNA sample independently measured for three times. Transcripts were quantified by TaqMan® Gene Expression Assays for Gja1 (4331182, Assay ID: Mm00439105_m1, Thermo Fisher Scientific, Waltham, MA) on a model 7300 Real-Time PCR system (Thermo Fisher Scientific, Waltham, MA). The real-time PCR was cycled at 95°C for 20 s, 40 cycles of 95°C for 1 s, and 60°C for 20 s. Mouse glyceraldehyde-3-phosphate dehydrogenase (GAPDH) was used as an endogenous control. Quantitative PCR analysis was performed as per the guidelines provided by Applied Biosystems using the comparative cycle threshold method.

### Western Blot Analysis

Total 400 μg protein from strial tissue of control, scrambled siRNA treated, and Gja1 siRNA treated groups was extracted in ice cold ACSF containing Halt™ Protease Inhibitor (1:100; 78425, Thermo Fisher Scientific, Waltham, MA) and transferred to cold RIPA Lysis and Extraction Buffer (89900, Thermo Fisher Scientific, Waltham, MA) with the protease inhibitor. A TissueRuptor (Kimble™ Kontes™ Pellet Pestle™; 11800104, Thermo Fisher Scientific, Waltham, MA) was used to homogenize tissue samples, which were then centrifuged for 15 min at 13,000 rpm. Concentration of proteins in the supernatant was measured using a UV-visible spectrophotometer (NanoDrop 1000, Thermo Fisher Scientific, Waltham, MA). A total of 120 μg protein from each cohort was loaded to lanes of a 10% sodium dodecyl sulfate-polyacrylamide gel to detect Cx43. Proteins were electrophoretically transferred to PVDF membranes and blocked with non-fat milk for 1 h at room temperature. Membranes were then incubated in primary antibody, a rabbit polyclonal anti-Cx43/GJA1 antibody (ab11370, Abcam, Cambridge, MA), or GAPDH (sc-32233, Santa Cruz Biotechnology, Dallas, TX) at a dilution of 1:1000 in skim milk overnight at 4°C. After three washes with TBST, the membranes were incubated for 1 h with the secondary antibody goat anti-rabbit IgG (H + L)-HRP (170-6515, Bio-Rad Laboratories, Hercules, CA) at a dilution of 1:3000 for 1 h at room temperature, and antigens were assessed using ECL Plus Western Blotting Detection Reagents (RPN2132, Amersham Biosciences, Piscataway, NJ). Image J (NIH, 1.52p) was used to quantify samples in the western blot analysis. All image panels presented in the manuscript were constituted from individual images processed and labeled using Adobe Photoshop (version CC 2017) with a resolution of 300 dpi. To enhance visualization, some of the images were equivalently enhanced after composition.

### Immunolabeling of Tight Junction-Associated Proteins in Primary Cell Lines and Isolated Strial Capillaries

Around 2 ml of purified ECs at passage 3 (at a density of 3.0 × 10^5^ ml) was seeded and grown on 35-mm collagen I-coated glass-bottom dishes in a growth medium for ~5 days. Cells were then transfected with Gja1 siRNA for 48 h, following the manufacturer’s guidelines. For negative control, the cells were transfected with scrambled siRNA. For the transfection, 10 μl TransIT-TKO® Transfection Reagent (MIR 2154, Mirus Bio LLC, Madison, WI) was mixed with 250 μl serum-free medium in a sterile tube, and 25 nM siRNA was added to the mixture. After incubation for 15 min at room temperature, the TransIT-TKO Reagent/siRNA mixture was added to the cell culture dish with 2.25 ml of fresh complete growth medium. The cells were then fixed in 4% PFA (pH 7.2) for 15 min at room temperature and washed in 2 ml of PBS (three times for 10 min). The cells were permeabilized in 0.25% Triton X-100 for 5 min at room temperature, washed with 2 ml PBS (three times for 10 min), and incubated with an immunofluorescence blocking solution at room temperature for 1 h. The cells were incubated overnight at 4°C with primary antibodies, ZO-1 Polyclonal Antibody (61-7100, Thermo Fisher Scientific, Waltham, MA), Anti-VE cadherin antibody (ab33168, Abcam, Cambridge, MA), Anti-Occludin antibody (ab31721, Abcam, Cambridge, MA), and Anti-Connexin 43/GJA1 antibody (ab78055, Abcam, Cambridge, MA; Table 1) in 1% BSA in PBS. After three washes in PBS for 10 min, the cells were incubated with the secondary antibody, Alex Fluor 568-conjugated goat anti-rabbit IgG (H + L) and Alex Fluor 488 goat anti-mouse IgG (H + L; A-11001, Thermo Fisher Scientific, Waltham, MA), diluted at 1:100 in 1% BSA in PBS solution for 1 h at room temperature. The cells were washed in 2 ml PBS (three times for 10 min) and imaged under FV1000 Olympus laser-scanning confocal microscope (Olympus FV1000, Japan). For capillary isolation, we used a previously reported “sandwich-dissociation” method for isolation (Yang et al., 2011; Zhang et al., 2012). Specifically, the cochleae from control (scrambled siRNA) and siRNA transfection groups were dissected after cardiovascular perfusion with saline, removed rapidly, and placed in a Petri dish filled with a hysiological solution containing 125 mmol/L NaCl, 3.5 mmol/L KCl, 1.3 mmol/L CaCl_2_, 1.5 mmol/L MgCl_2_, 0.51 mmol/L NaH_2_PO_4_, 10 mmol/L Hepes, and 5 mmol/L glucose at pH 7.4 with osmolarity adjusted to 310 mmol/kg. The stria vascularis was peeled away from the spiral ligament gently with Dumont tweezers (110 mm, 0.1 × 0.06 mm tip) and a Tungsten Dissecting Probe (50 mm, 0.5-mm diameter rod) under an Olympus SZ61 dissecting microscope. The stria vascularis was placed in a glass-bottomed microwell dish (dish diameter: 35 mm; microwell diameter: 10 mm; coverglass: 0.16–0.19 mm; MatTek Corporation) filled with the physiological solution. A glass coverslip (0.16 mm) then was positioned over the stria vascularis, and the tissue was sandwiched gently between the glass surfaces. Gentle pressure was applied to compress the stria vascularis against the two glass surfaces. By repeating this step, the nonvascular tissues were dispersed into the solution and separated from the capillaries. Nonvascular cells were flushed away gently with a 100-μl micropeptide; the clean microvessels were adhered and “printed” onto the bottom of the dish, analogous to the offset-printing deposit of a pattern of ink onto paper. The isolated capillaries were then immunolabled with ZO-1 Polyclonal Antibody (61-7100, Thermo Fisher Scientific, Waltham, MA), Anti-VE cadherin antibody (ab33168, Abcam, Cambridge, MA), and Anti-Occludin antibody (ab31721, Abcam, Cambridge, MA; Zhang et al., 2012). The samples were imaged under the Olympus laser-scanning confocal microscope.

### Assessment of Vascular Permeability *in vivo*

Vascular permeability in the scrambled siRNA treated and Gja1 siRNA treated groups was assessed repeatedly using a albumin-fluorescein isothiocyanate (FITC) conjugate tracer (A9771, FITC-albumin, 66 kDa, Sigma, St. Louis, MO) and a fluorescein isothiocyanate-dextran tracer (FD10S, FITC-dextran, 10 kDa, Sigma, St. Louis, MO). Around 100 μl tracers (40 mg/ml) were intravenously administered to the tail vein of anesthetized control siRNA treated and Gja1 siRNA treated groups for 30 min prior to transcardial perfusion of the mice with PBS. After circulating blood cells were completedly flushed away, the stria vascularis was gently emoved. The tissues were then homogenized in 1% Triton X-100 in PBS, with the lysate centrifuged at 13,000 rpm for 20 min. The fluorescence signal, reflecting concentration of fluorescence tagged tracer trapped in the stria vascularis, was detected with a Tecan GENios Plus microplate reader at an excitation wavelength of 450 nm, with the emission acquired through a 560-nm filter.

### Permeability Assay

Purified ECs were seeded on polyethylene terephthalate membranes of Transwell inserts (353095, Corning, New York, NY) in 24-well plates at 10^5^ cells per insert and cultured for 3 days to form a complete monolayer, as previous reported (Zhang et al., 2015). The cells were then transfected with Gja1 siRNA or scrambled siRNA for 48 h (at a 25 nM final concentration), as per TransIT-TKO guidelines. Endothelial monolayer permeability was assessed by determining the flux of FITC-dextran in the upper and lower chambers. FITC-dextran (10 kDa, 10 mg/ml) was added to the insert at a final concentration of 1 mg/ml. After 20 min of incubation at room temperature, 100 μl medium was removed from the well of the Receiver Tray and transferred to a 96-well plate for measurements. The permeability of the EC monolayer was measured by detecting FITC-dextran fluorescence using a fluorescence plate reader with filters appropriate for 485 nm excitation and 535 nm emission (Zhang et al., 2012).

### Statistics

SPSS (18.0) software was used for the data analysis. For determining sample size, we used power analysis to calculate the minimum sample size for a power of 0.8. We used the “resource equation” method when calculating sample size for ANOVA tested data. Both the Shapiro–Wilk test and Q-Q plot were used to test data distribution. Data were presented as means ± SD. Since we took random samples of each group, the data are independent. Student’s *t*-test was used for comparison of two groups and one-way ANOVA was used for comparison of three groups. *p* < 0.05 was considered statistically significant.

## Results

### Cx43 Immunoreactivity Was Detected in the Cochlear Stria Vascularis, and in a Co-culture of PVM-EC Primary Cell Lines

**Figure 1:**
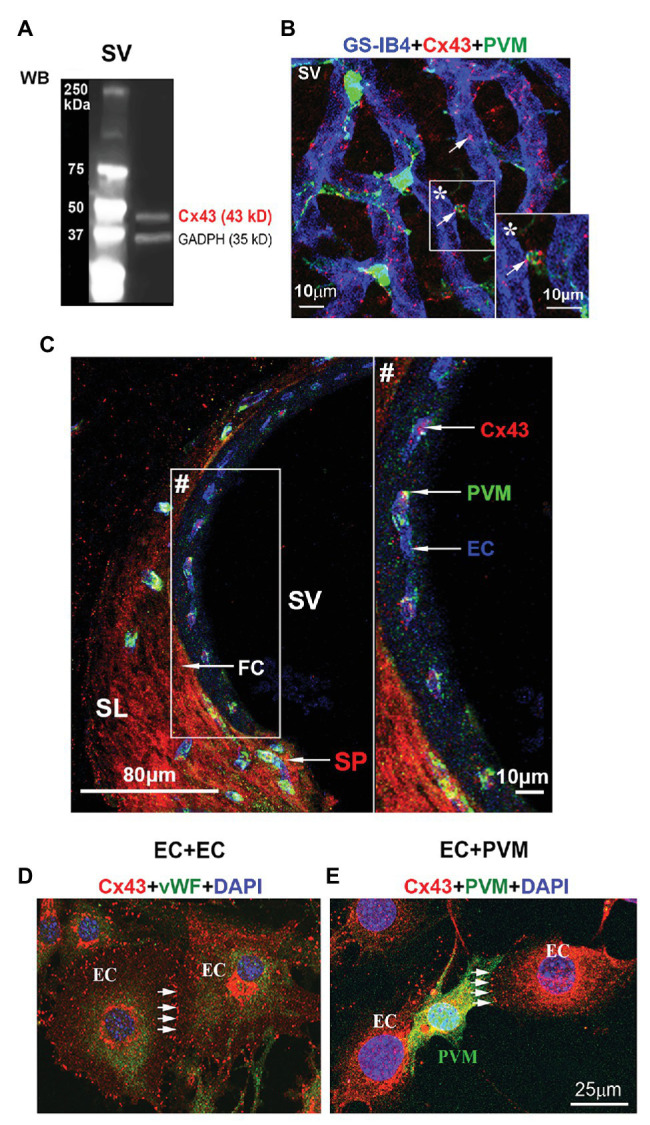
Connexin 43 (Cx43) immunoreactivity detected in the stria vascularis and spiral prominence (SP), and in primary cell lines. **(A)** Western blot analysis shows Cx43 protein tissue expression in the stria vascularis. **(B)** The confocal image of triple immunolabeled cells using antibody for Cx43 to show Cx43-positive spots predominately expressed in blood vessels [labeled with Griffonia simplicifolia IB4 (GS-IB4) conjugated to Alexa Fluor 647] and end-feet of PVMs^GFP^ (green) in a whole-mount surface preparation of the stria vascularis. **(C)** The confocal projection image shows that Cx43 in the SP (SP/arrow) is primarily expressed in endothelial cells (ECs/arrow) and fibrocytes (FCs/arrow) in a cross-section preparation of the cochlear lateral wall. **(D)** The confocal projection image further shows Cx43 expression at the contact border between ECs labeled with antibody for von Willebrand factor (green), an EC marker protein. **(E)** The confocal projection image shows Cx43 expression at the contact border between ECs and PVMs^GFP^ (green). SV, stria vascularis; SL, spiral ligament; SP, spiral prominence; PVM, perivascular resident macrophage; EC, endothelial cell; FC, fibrocyte. ^*^ is the magnified insert image of Cx43 expression in the EC-PVM contacts. ^#^ is the zoomed image of Cx43 expression from an insert in **panel (C)**.

CX43, a GJ protein, has been identified widely distributed in the cardiac and neural system (Hansen et al., 2014). In this study, using western blot, we detected Cx43 immunoreactivity in adult mouse stria vascularis (Figure 1A). With immunofluorescence labeling in combination with confocal microscopy, we found that Cx43 was expressed predominantly in capillary ECs and in the contacts between GFP-tagged PVMs (PVMs^GFP^) and ECs in whole-mounted stria vascularis (Figure 1B). Cx43 expression in the EC-PVM contacts is particularly evident in the magnified insert image (*) in Figure 1B. Cx43 expression was also seen in the spiral ligment (SL), particularly in fibrocytes (FCs) of the spiral prominence (SP), as shown in a cross-section of the cochlear lateral wall (Figure 1C). Consistent with what we see in a whole-mounted surface preparation, Cx43 expression in ECs and PVMs is further demonstrated in the cross-section preparation. The Cx43 expression is better visualized in the zoomed image (#) from an insert (#) in panel C. Consistent with Cx43 expression found in strial ECs and PVMs of whole-mounted tissue, we also detected Cx43 expression in co-culture of primary cell lines of strial ECs and PVMs. Under confocal microscopy, we found Cx43 particularly expressed in EC-EC contacts (Figure 1D) of an EC cultured model and in PVM-EC contacts (Figure 1E) of an EC + PVM co-culture model. The results indicate that Cx43 is expressed in strial BLB component cells of adult cochlea.

### Cx43 Mediation of PVM-EC Communication Tested in an *in vitro* Primary Cell Line Co-culture Model

**Figure 2:**
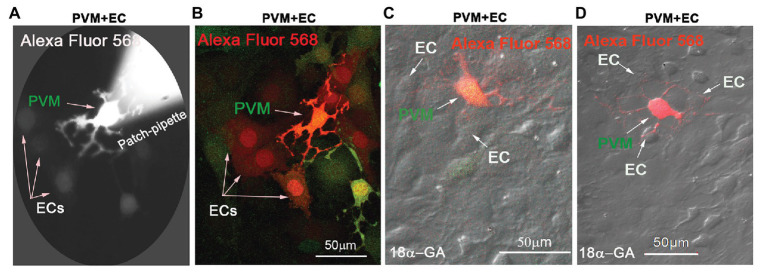
Cx43 mediates intercellular communication between PVMs and ECs in an *in vitro* co-culture model. **(A)** Alexa Fluor 568 dye communication between an injected PVM and nearby ECs 1 min after dye loading under epi-fluorescence microscopy. **(B)** The same experiment was recorded under confocal fluorescence microsopy to better show the injected fluorescent Alexa Fluor 568 dye has spread to surrounding ECs within 5 min of PVM^GFP^ dye injection (green). Arrows indicate dye communication with the cells. **(C,D)** DIC images are overlaid on confocal fluorescence images to show that the injected Alexa Fluor 568 dye was restricted to PVMs (red/green), not transferred to surrounding ECs, when Cx43 was blocked with 18α-Glycyrrhetinic acid (18α-GA).

In this study, we found Cx43 GJs actively involved in intercellular communication between ECs and PVMs in an *in vitro* model. PVMs encoded with GFP were co-cultured with ECs for 2 consecutive days. After the ECs and PVMs established connections (day 3), Alexa Fluor 568 fluorescence dye was delivered to the PVM^GFP^ by a patch-clamp pipette. We identified PVMs by the green fluorescence under an epi-fluorescence microscope. One minute later, we detected that the injected fluorescence dye had propagated to surrounding cells, which included connected ECs and nearby PVMs^GFP^. Figure 2A is a representative image showing dye diffusion 1 min after the dye was injected into the PVMs^GFP^ under an epi-fluorescence microscope. Figure 2B is the same experiment but recorded under a confocal fluorescence microscope 5 min after the dye was injected to the PVMs. The confocal image enables us to better display the dye communication between the PVMs and three connected ECs and one neighboring PVM.

To determine whether the dye communication between PVMs^GFP^ and ECs involved Cx43 GJ protein, we employed a GJ blocker, 18α-GA, which has been reported to block Cx43 mediated intercellular communication (Zhu et al., 2016; Jiang et al., 2017). As shown in Figures 2C,D, we found that blocking GJ with 18α-GA significantly blocked dye transfer from an injected PVM^GFP^ to the connected ECs. These results suggest that Cx43 mediates intercellular communication between PVMs and ECs.

### Downregulation of Cx43 Expression in the Adult Mouse Cochlea Decreases the Endocochlear Potential

**Figure 3:**
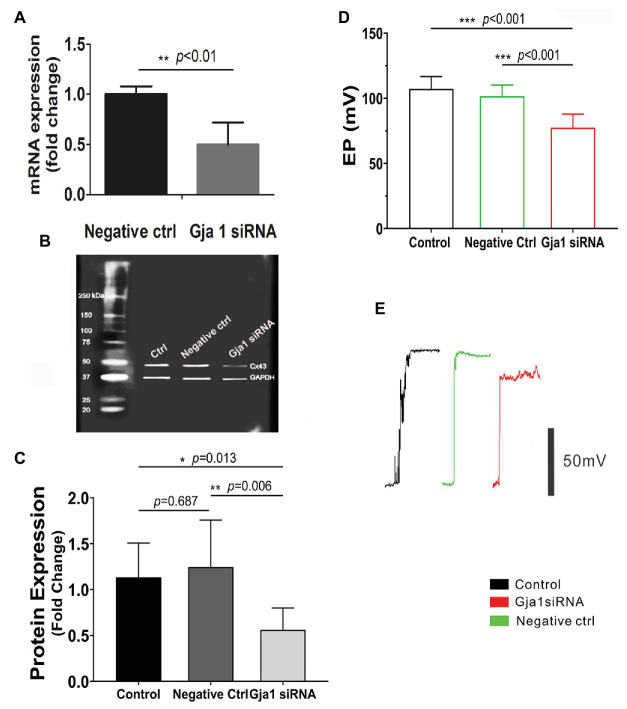
Downregulation of Cx43 reduces endocochlear potential (EP). **(A)** Real-time PCR analysis shows that Cx43 messanger RNA (mRNA) expression is significantly decreased in the stria vascularis of Gja1 small interfering RNA (siRNA) treated groups [*n*_negative ctrl_ = 6, *n*_Gja1 siRNA_ = 6, *t*(6.186) = 5.327, ^**^p_(negative ctrl vs. Gja1 siRNA)_ < 0.01, Student’s *t*-test]. **(B,C)** Western blot analysis shows that Cx43 protein expression is significantly reduced in the Gja1 siRNA treated group [*n* = 7, *F*(2,18) = 5.859, ^*^p = 0.011, *p*_(ctrl vs. negative ctrl)_ = 0.687, ^*^p_(ctrl vs. Gja1 siRNA)_ = 0.013, ^**^p_(negative ctrl vs. Gja1 siRNA)_ = 0.006, one-way ANOVA]. **(D)** Average EP in the control, control scramled siRNA treated, and Gja1 siRNA treated group. A slight but significant decrease in Cx43 protein expression is seen in the Gja1 siRNA treated group, compared to the normal and control groups [*n* = 6, *F*(2,21) = 22.045, ^***^p < 0.001, one-way ANOVA, ^***^p_(ctrl vs. Gja1 siRNA)_ < 0.001, ^***^p_(negative ctrl vs. Gja1 siRNA)_ < 0.001, mean ± SD]. **(E)** Representative EP waveform in the control, control siRNA treated, and Gja1 siRNA treated group.

The EP is generated by electrogenic secretion of potassium-rich endolymph from the stria vascularis (Patuzzi, 2011). In this study, EP was measured from the scala media by micropipette through it in control siRNA treated and Gja1 siRNA treated mice. Gja1 siRNA (40 μM) at the same concentration of scrambled siRNA (control siRNA) was administered to the cochlea *via* the middle ear cavity for 3 days. Real-time PCR analysis showed that the level of Cx43 messenger RNA (mRNA) in the Gja1 siRNA treated animals was significantly lower than in the control siRNA treated animals (*n* = 6, ^**^p < 0.01, Figure 3A). Cx43 protein levels were consistently lower in the Gja1 siRNA treated group (analyzed by WB) than in the control siRNA treated-group [*n* = 7, ^*^p = 0.013, one-way ANOVA, ^**^p_(negative ctrl vs. Gja1 siRNA)_ = 0.006, Figures 3B,C]. We found that the EP was significantly affected by downregulation of Cx43 expression. In the control group, an average EP value is ~ + 107 ± 9.5 mV. In contrast, the EP in Gja1 siRNA treated animals was reduced to ~ + 77 ± 10.4 mV. Figures 3D,E demonstrate average EP values and representative EP waveforms in control, siRNA, and scrambled siRNA treated-groups. Our data indicate that normal Cx43 expression is essential for a normal EP.

### Downregulation of Cx43 Expression in the Adult Mouse Cochlea Causes Mild Hearing Loss

**Figure 4:**
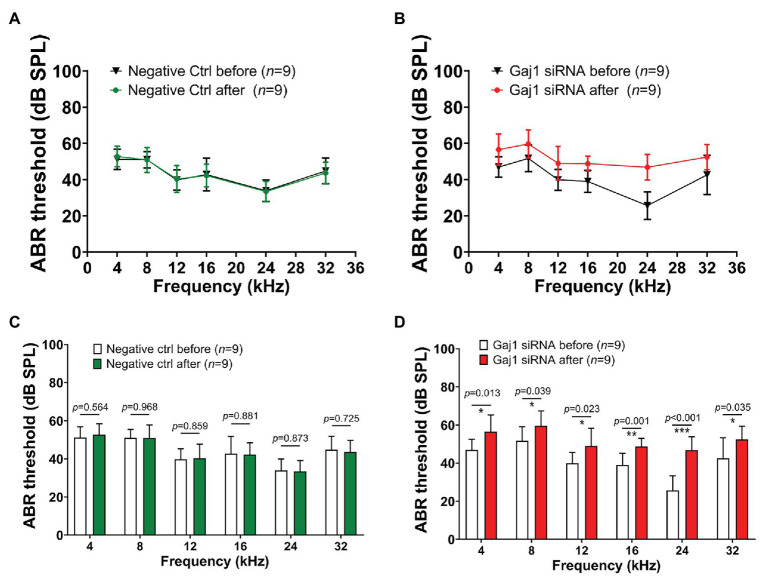
Downregulation of Cx43 expression causes hearing loss. **(A,C)** No elevated hearing threshold was detected at any of the tested frequencies in the control siRNA treated groups (*n* = 9, *p* > 0.05, Student’s *t*-test, mean ± SD). **(B,D)** Downregulation of Cx43 caused increased hearing threshold at 4 kHz [*n* = 9, *t*(16) = −2.802, ^*^p = 0.013, Student’s *t*-test, mean ± SD], 8 kHz [*n* = 9, *t*(16) = −2.243, ^*^p = 0.039, Student’s t-test, mean ± SD], 12 kHz [*n* = 9, *t*(16) = −2.507, ^*^p = 0.023, Student’s *t*-test, mean ± SD], 16 kHz [*n* = 9, *t*(16) = −3.966, ^**^p = 0.001, Student’s *t*-test, mean ± SD], 24 kHz [*n* = 9, *t*(16) = −6.068, ^***^p < 0.001, Student’s *t*-test, mean ± SD], and 32 kHz [*n* = 9, *t*(16) = −2.307, ^*^p = 0.035, Student’s *t*-test, mean ± SD].

Hearing sensitivity is also affected by downregulation of Cx43 expression. We found that no significant elevation in hearing threshold was detected in the control siRNA treated-group (Figures 4A,C). In contrast, the hearing threshold in the Gja1 siRNA treated-mice was significantly elevated across all tested sound frequencies. Figures 4B,D show the respective elevation of hearing threshold at different sound frequencies after Gja1 siRNA treatment at 4 kHz (*n* = 9, ^*^p = 0.013), 8 kHz (*n* = 9, ^*^p = 0.039), 12 kHz (*n* = 9, ^*^p = 0.023), 16 kHz (*n* = 9, ^**^p = 0.001), 24 kHz (*n* = 9, ^***^p < 0.001), and 32 kHz (*n* = 9, ^*^p = 0.035). Our data indicate that reduced Cx43 expression in the cochlea causes mild hearing loss across the spectrum from low frequency to high frequency.

### Downregulation Cx43 Expression Increases Strial Vascular Leakage *in vivo* and *in vitro*

**Figure 5:**
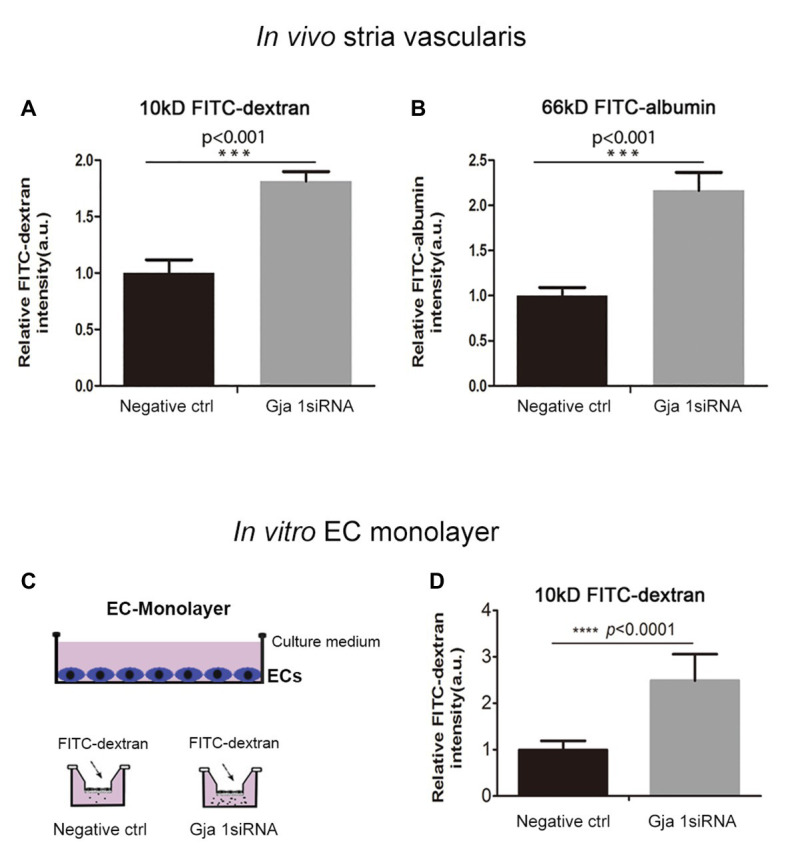
Suppression Cx43 increases vascular leakage *in vivo* and *in vitro*. **(A,B)** More fluorescein isothiocyanate (FITC)-dextran [MW 10 kDa; *n*_ctrl_ = 3, *n*_Gja1 siRNA_ = 3, *t*(4) = −9.797,^***^p_(ctrl vs. Gja1 siRNA)_ < 0.001, Student’s *t*-test] and FITC-albumin [MW 66 kDa; *n*_ctrl_ = 3, *n*_Gja1 siRNA_ = 3, *t*(4) = −9.134, ^***^p_(ctrl vs. Gja1 siRNA)_ < 0.001, Student’s *t*-test] leakage was detected in Gja1 siRNA-treated animals than in control scrambled siRNA treated animals. **(C)** An illustration of the EC monolayer model. **(D)** Significantly increased permeability is found in the EC-monolayer of the Cx43 downregulated group compared to the control EC-monolayer group [*n*_ctrl_ = 12, *n*_Gja1 siRNA_ = 12, *t*(13.404) = −8.626, ^****^p_(ctrl vs. Gja1 siRNA)_ < 0.0001, Student’s *t*-test, mean ± SD].

What mechanisms might explain the drop in EP and hearing loss when Cx43 expression is suppressed? It is known that direct intercellular communication *via* GJs is critical for control and coordination of blood barrier function (Figueroa and Duling, 2009; Ivanova et al., 2017). In the brain, the absence of Cx43 weakens the BBB (Ezan et al., 2012). Does suppression of Cx43 likewise weaken the BLB? In this study, we investigated the effect of suppressing Cx43 expression on BLB integrity. Vascular permeability was assessed in both *in vitro* in an EC cell line based model and *in vivo*. In the *in vivo* animal model, the vascular permeability in control and Gja1 siRNA treated animals was assessed using an FITC-conjugated dextran tracer. Low molecular weight 10 kDa FITC-dextran or medium molecular weight 66 kDa FITC-albumin was administered intravenously to the mice at a dose of 40 mg/ml in 100 μl physiological solution for 30 min prior to tissue harvest. FITC-dextran leakage was quantified using a leakage index defined in previous publications (Zhang et al., 2015). Our results showed that downregulation of Cx43 increases BLB permeability, as shown in Figures 5A,B (*n* = 3; ^**^p < 0.001).

To corroborate our *in vivo* findings, we constructed an *in vitro* endothelial monolayer model (Figure 5C), in which a cochlear primary EC cell line was seeded on Transwell filters to form a monolayer (Zhang et al., 2015) and treated with Gja1 siRNA for 48 h. Endothelial monolayer permeability was assessed by determining the flux of FITC-dextran in the lower chambers. The endothelial monolayer barrier was demonstrated to be more permeable in the Gja1 siRNA treated groups, shown in Figure 5D than in the control siRNA treated group (*n* = 12, ^****^p < 0.0001).

### Downregulation Cx43 Expression Decrease Expression of Tight Junction *in vivo* and *in vitro*

**Figure 6:**
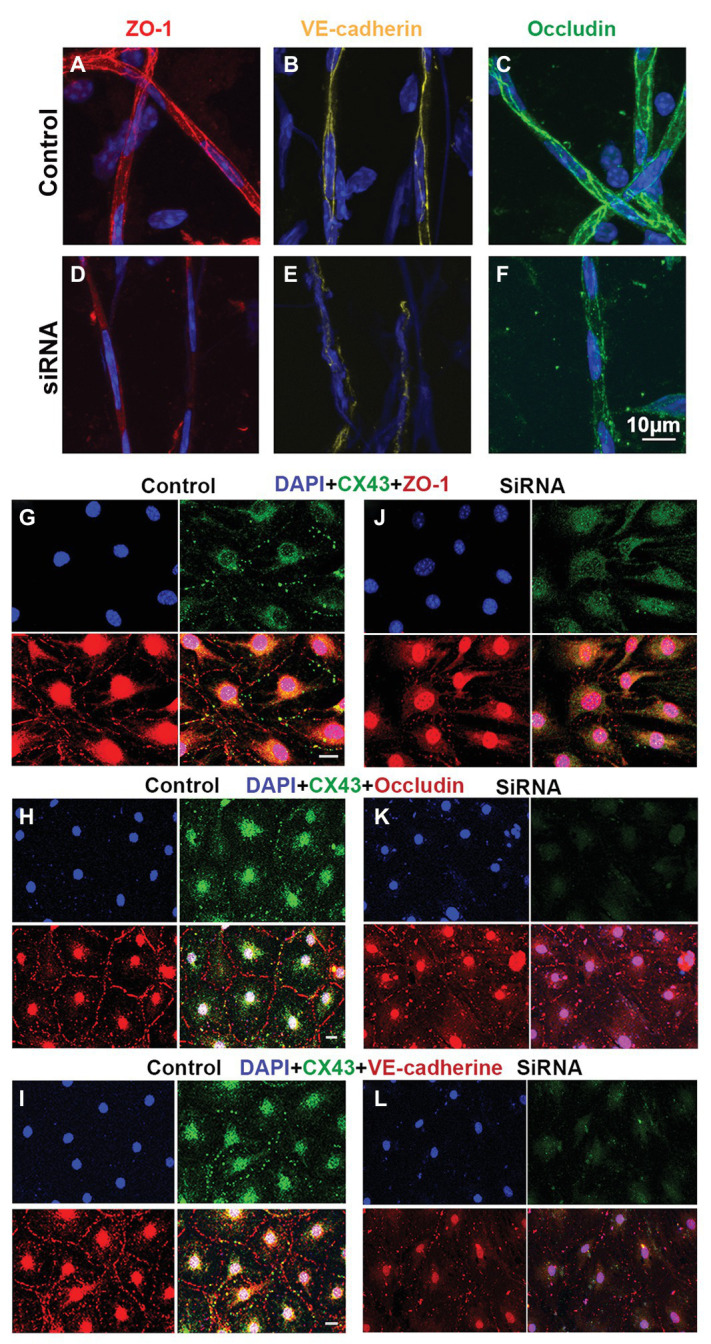
Suppression of Cx43 alters the TJ distribution pattern in EC monolayers. **(A–F)** Tight junction (TJ) protein (ZO-1, VE-cad, and occludin) expression patterns in isolated capillaries of control **(A–C)** and Gja1 siRNA-treated groups **(E–F)**. Gja1 siRNA-treated groups show reduced suppression of ZO-1, VE-cad, and occludin. **(G–I)** TJ protein (ZO-1, VE-cad, and occludin) expression patterns in the control group. **(J–L)** TJ protein (ZO-1, VE-cad, and occludin) expression patterns in the Cx43 suppressed groups. Downregulation of Cx43 had a discernable effect on the distribution pattern of TJ proteins (scale bar: 10 μm).

The permeability of the blood barrier is largely a function of the tightness of the intercellular junction. The major TJ-associated proteins in the barrier are occludin, various claudins, ZO-1, and adherens-junction proteins (Jiao et al., 2011; Campbell et al., 2017). Several tight- and adherens-junction proteins, including ZO-1, occludin, and VE-cadherin, have been found in the strial BLB (Zhang et al., 2012, 2015; Shi, 2016). In this study, relative to expression of ZO-1, occludin, and VE-cadherin in the capillaries under normal conditions (Figures 6A–C), we found that expression of these proteins in the endothelial tube is clearly changed at day 3 after siRNA treatment *in vivo* (Figures 6D–F). Connsistent with the findings seen *in vivo*, TJ protein distribution in the EC monolayer was dramatically altered within 48 h in the siRNA treated groups *in vitro*. In controls, TJs are prominent, outlining the borders of connected ECs throughout the monolayer, as shown in Figures 6G–I. In contrast, downregulation of Cx43 leads to randomly scattered, irregular clusters, as shown in Figures 6J–L.

## Discussion

The GJ protein, Cx43, encoded by the GJA1 gene, is the most abundant connexin in the cardiovascular system. Cx43 is a crucial factor in stabilizing electrical conduction in cardiac muscle and maintaining the integrity of the (BBB; Nagy and Rash, 2000; Boulay et al., 2016). Cx43 is pervasive in the inner ear as well. Mutation of Cx43 was found by Yang, the second most common mutation (27.45%) in the inner ear causing genetic sensorineural hearing loss, after Cx26 mutation causing 45.16% of genetic sensorineural hearing loss (Yang et al., 2007). The mechanism by which Cx43 mutation causes hearing loss is not fully understood. In this study, we used immunocytochemical techniques to examine normal Cx43 expression in the BLB of the stria vascularis. Cx43 expression was found in strial ECs and PVMs in adult C57BL/6 mouse cochlea. In particular, we found extensive Cx43 expression in the contacts between PVMs and ECs. Consistent with Cx43 expression *in situ*, we also found Cx43 expression in co-cultured primary cell lines of strial ECs and PVMs. Using a patch clamp technique, we demonstrated functional communication between PVMs and ECs. When GJs were treated with a GJ blocker, dye communication between the PVMs and ECs was blocked. Suppression of Cx43 with siRNA *in vivo* significantly increased hearing threshold and also caused the EP to drop. Further study showed suppression of Cx43 to markedly affect BLB integrity and increase BLB permeability. Our data indicate that Cx43 is essential for strial BLB integrity, EP, and hearing sensitivity in the adult ear.

Previous studies from different labs have reported detection of Cx43 expression in various cells of the cochlea, including in the organ of Corti, lateral wall, spiral ganglion neurons (SGNs), and their neurite terminals innervating inner and outer HCs (Suzuki et al., 2003; Liu and Yang, 2015). However, reports on the distribution of Cx43 in the cochlea are inconsistent. Most data have shown a broad distribution of Cx43 in different cochlear regions in the early stages of cochlear development, especially for synaptogenesis and establishment of auditory neurotransmission (Liu and Yang, 2015). However, a few groups have shown Cx43 expression in spiral ganglia to persist into adulthood (Liu and Yang, 2015). For example, Kim et al. (2013) reported that Cx43 robustly presents in retro-cochlear centers in adult CBA/J mice. Liu et al. (2014) showed Cx43 expression in satellite glial cells (SGCs) in adult human and guinea pig. These satellite cells play an important role in protecting SGNs. We presume that the inconsistent reports from different laboratories might be due to differences in experimental staining procedure and animal species (Nagy et al., 2001).

Cx43 expression in the stria vascularis has only been reported in post-natal mice (P0, P14, or P17; Cohen-Salmon et al., 2004; Liu and Yang, 2015). For example, Suzuki et al. (2003) reported Cx43 expression in the adult stria vascularis of rat cochlea. They demonstrated Cx43-positive areas localized in the stria vascularis and spiral ligament. In the present study, we obtained results consistent with Suzuki, detecting a unique pattern of Cx43 distribution in the strial vascularis of adult mouse cochlea (as shown in Figure 1). Cx43 expression was typically expressed in contacts between PVM end-feet and the endothelium of blood vessels. We further verified Cx43 protein in the stria vascularis by western blot. Moreover, we demonstrated that Cx43 mediated intercellular communication occurs between ECs and PVMs in *in vitro* primary cell line models. These findings provide corroborating evidence in support of an early finding by Takeuchi and Ando (1998), in which they demonstrated that melanocytes, which we later identify as PVMs (Zhang et al., 2012; Neng et al., 2013), communicate and are dye-coupled with ECs through GJs *in vivo*. In this study, we also noticed less Cx43 expression in the vascular network of the spiral ligament (Figure 1C), further confirming our earlier conclusion that the two vascular networks are functionally distinct (Shi, 2011). The microvessels in the spiral ligament have roles in regulation of blood flow, as vessels in this region contain a large number of smooth muscle cells and contractile PCs (Shi et al., 2008). In contrast, strial microvessels have endothelium specialized to form a tight BLB, crucial for maintaining the EP, ion transport, and endolymphatic fluid balance, necessary for cochlear sensitivity.

GJs are critical for coordination of cell function, allowing passage of electrical charge between connected cells and exchange of chemical signals and energy substrates (Zhang et al., 2018). It is generally accepted that the stria vascularis in the cochlea produces the K^+^-rich endolymph, which is the driving force for sound transduction by sensory HCs (Zdebik et al., 2009). GJs are an important link in the intracellular K^+^-recycling pathway to the stria vascularis (Zdebik et al., 2009). The distinctive pattern of Cx43 distribution in strial BLB component cells and the functional communication mediated by Cx43 raises the question: Does Cx43 play a critical role in maintaining BLB integrity and EP generation? To investigate this question, we employed siRNA silencing to downregulate Gja1 in adult mice and suppress Cx43 expression. The downregulation caused the EP to drop, and, correspondingly, hearing loss was seen at some frequencies, particularly high frequencies. The patchy hearing loss could be due to the effective distribution of siRNA in the cochlea. Agents injected into the middle ear can form a concentration gradient from basal regions to the apex (Plontke et al., 2008). Consistent with the notion, we have frequently observed in the lab that delivery of chemicals to the middle ear mostly concentrates at the basal turn, although we have also found the efficacy of some chemicals on cochlear function higher at the apical turn, presumably due to chemical properties.

**Figure 7:**
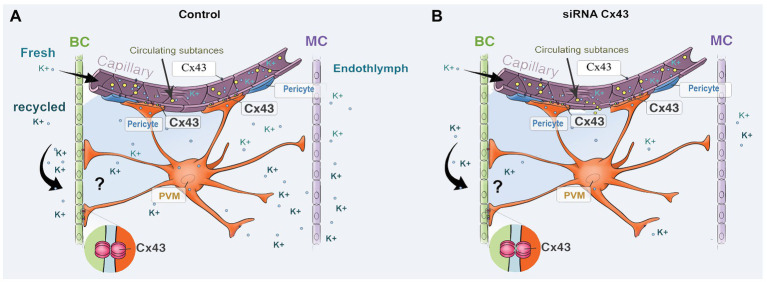
An illustration of the effect of Cx43 expression in endothelium and in PVMs on K^+^ movement and EP generation. **(A)** Perivascular resident macrophage (PVM; melanocyte) end-feet contact the capillary wall and also inter-digitally connect with BCs when CX43 is normally expressed. **(B)** siRNA downregulates the expression of CX43, affecting both circulating and recycled K^+^, reducing the EP. Downregulation of Cx43 also causes increased BLB permeability, leading to an intrastrial electric shunt which also reduces the EP.

The strial BLB is normally one of the tightest blood-tissue barriers in mammals and is critical for maintaining cochlear homeostasis, particularly for maintaining the EP. Break in the barrier could result in an intrastrial electric shunt (Cohen-Salmon et al., 2007). In this study, we presume Cx43 expression in endothelium and PVMs together with expression of other GJ proteins, including Cx26 and Cx30, affect K^+^ movement and the EP, as shown in Figure 7. PVMs are interdigitated between marginal and basal cell processes (Steel and Barkway, 1989) and surrounding blood vessel walls (Shi, 2016). We hypothesize the Cx43 expressed in the PVMs facilitates K^+^ transport for maintaining the EP through two pathways: (1) circulating blood (“fresh” K^+^ restores K^+^ depleted in recycling) and (2) K^+^ returned through epithelial-syncytium layers (recycled K^+^? as illustrated in Figure 7). In the first pathway, K^+^ may be taken up into capillary ECs from the blood stream through K^+^ channels or Na^+^/K^+^/Cl^−^ co-transporters expressed at the apical surface of ECs. The K^+^ is subsequently transmitted to PVMs *via* Cx43. In the second pathway, PVMs facilitate K^+^ recycling through GJs, including Cx43, expressed in the FCs and basal cells, as we identify in Figure 1C. Downregulation of Cx43 would interrupt the K^+^ recycling from either source to the intrastrial space and lead to an EP drop. In an earlier paper, we showed that normal PVMs maintain capillary integration by having a role in isolating the intrastrial space from the blood flow (Zhang et al., 2012, 2013). In the present study, we further demonstrate that downregulation of CX43 in ECs weakens BLB integrity (as shown in Figure 6) and can cause an intrastrial electric shunt. These would likely contribute to a drop in the EP as well.

In this study, we found that suppressed Cx43 expression causes increased vascular leakage in the strial BLB to low and medium size molecules, as demonstrated in Figure 5. Moreover, we found that downregulation of Cx43 had a discernable effect on the distribution pattern of occludins, ZO-1, and adherens-junction proteins such as VE-cadherin. These proteins are essential for the structural and functional integrity of the strial BLB (Shi, 2016). In controls, TJs line the borders of contacted ECs, as shown in Figure 6. In contrast, with downregulation of Cx43, we see clusters or randomly scatted distributions of TJs along EC-EC contacts. Consistent with our *in vivo* findings that suppression of Cx43 increases strial BLB permeability, downregulation of Cx43 *in vitro* also made the EC monolayers more permeable. Our results are consistent with recent reports on non-auditory systems, in which Cx43 affects TJ formation and loss of Cx43 leads to blood barrier hyper-permeability (Hollenbach et al., 2018; Johnson et al., 2018; Bazzoun et al., 2019). The mechanism by which suppression of Cx43 alters TJ organization was not determined in this study, and, in fact, the mechanism by which suppression of Cx43 alters TJ organization is largely unknown. Some studies have shown that Cx43 hemichannels contribute to the assembly of cell junctions through modulation of the intracellular oxidative status (Chi et al., 2016). Li et al. (2010) has shown that regulation of Cx43 by siRNA can disassemble adhesive assemblies. Other studies have shown that specific domains of Cx43, such as the ser(9) and ser(10) at the C-terminal, serve as binding sites for interaction with various proteins, including ZO-1 (Xiao et al., 2011). A further study from Laing et al. (2005) has shown that the Cx43 has interactions with TJ proteins such as ZO-1 to form lipid rafts in GJ plaques. Taken together, these studies underscore the functional dependence between TJ and GJ proteins. However, further study is needed to fully elucidate the mechanisms by which Cx43 affects expression of TJ protein in the strial BLB. Normal blood flow to the ear is critical for cochlear homeostasis and extremely important for generating the EP on which sound transduction by HCs depends (Ohlemiller et al., 2008). The EP drop could also reflect changes in blood supply due to the downregulation of Cx43.

Taken together, our data confirm Cx43 expression in the strial BLB and spiral ligament of the adult cochlea. Suppression of Cx43 leads to BLB hyper-permeability, decrease in EP, and loss of hearing sensitivity.

## Data Availability Statement

All datasets presented in this study are included in the article/supplementary material.

## Ethics Statement

All procedures in this study were reviewed and approved by the Institutional Animal Care and Use Committee (IACUC) at Oregon Health & Science University (IP 00000968).

## Author Contributions

JZ, XW, ZH, and LN were involved in all aspects of the experiments. Specifically, JZ, ZH, and LN did the immunohistochemistry, RT-PCR, WB, and ABR test. In addition, ZH and XW performed the EP measurement. JZ and LN performed the *in vitro* cell line study and were involved in data analysis. JZ, ZH, and YZ formatted the references. XS supervised the experiments and wrote the manuscript. All authors contributed to the article and approved the submitted version.

### Conflict of Interest

The authors declare that the research was conducted in the absence of any commercial or financial relationships that could be construed as a potential conflict of interest.

## Nomenclature

EP: Endocochlear potential
ABR: Auditory brainstem response
PVM: Perivascular resident macrophage.
